# Patient and Health Care Provider Experiences With a Recently Introduced Patient Portal in an Academic Hospital in the Netherlands: Mixed Methods Study

**DOI:** 10.2196/13743

**Published:** 2019-08-20

**Authors:** Maria M T Vreugdenhil, Sander Ranke, Yvonne de Man, Maaike M Haan, Rudolf B Kool

**Affiliations:** 1 IQ healthcare Radboudumc Nijmegen Netherlands

**Keywords:** patient portals, patient access to records, patient participation, professional-patient relations

## Abstract

**Background:**

In the Netherlands, the health care system and related information technology landscape are fragmented. Recently, hospitals have started to launch patient portals. It is not clear how these portals are used by patients and their health care providers (HCPs).

**Objective:**

The objective of this study was to explore the adoption, use, usability, and usefulness of a recently introduced patient portal in an academic hospital to learn lessons for the implementation of patient portals in a fragmented health care system.

**Methods:**

A mixed methods study design was used. In the quantitative study arm, characteristics of patients who used the portal were analyzed, in addition to the utilization of the different functionalities of the portal. In the qualitative study arms, think-aloud observations were made to explore usability. Focus group discussions were conducted among patients and HCPs of the dermatology and ophthalmology outpatient departments. Thematic content analysis of qualitative data was carried out and overarching themes were identified using a framework analysis.

**Results:**

One year after the introduction of the portal, 24,514 patients, 13.49% of all patients who visited the hospital, had logged in to the portal. Adoption of the portal was associated with the age group 45 to 75 years, a higher socioeconomic status, and having at least one medical diagnosis. Overarching themes from the qualitative analyses were (1) usability and user-friendliness of the portal, (2) HCP-patient communication through the portal, (3) usefulness of the information that can be accessed through the portal, (4) integration of the portal in care and work processes, and (5) HCP and patient roles and relationships.

**Conclusions:**

One year after the introduction of the patient portal, patients and HCPs who used the portal recognized the potential of the portal to engage patients in their care processes, facilitate patient-HCP communication, and increase patient convenience. Uncertainties among patients and HCPs about how to use the messaging functionality and limited integration of the portal in care and work processes are likely to have limited portal use and usefulness.

## Introduction

### Background

Patient portals are promising tools to support patient involvement and patient-centered care [1-3]. Some of the functionalities of patient portals are primarily aimed at increasing patients’ convenience, for example, the functionalities for scheduling appointments or medication reconciliation [4,5]. Other functionalities provide patients with relevant information about their health and offer access to general medical information, such as clinical guidelines, or to personal health information in their medical records [4]. In addition, many patient portals can be used for secure messaging to facilitate communication between patients and health care providers (HCPs) [4].

In the United States, patient portals are generally owned and administered by health institutions such as hospitals or health insurance companies together with their affiliated hospitals and clinics. In contrast, in Australia [1] and various European countries, for example, Denmark, Estonia, France [6], and, more recently, Finland [1] and Sweden [7], national patient portals have been launched. Beside these institution-based and national portals that are meant for the general population, there are also disease-specific patient portals that offer Web-based services for specific groups of patients, for example, for patients with diabetes or chronic kidney disease [8-11].

In the Netherlands, patient portals aimed at the general population are relatively new. In 2011, the development of a national patient portal was called off because of political resistance owing to privacy concerns. Since then, various health care organizations have started to develop institution-based patient portals. Academic hospitals have been leading in the implementation of hospital-based patient portals, using the options provided by the health information systems that they use. General hospitals have been following this trend [12]. Between 2014 and 2016, the percentage of medical specialists that provided their patients Web-based access to test results increased from 6% to 18%. In 2016, approximately 30% of the medical specialists indicated that patients could ask them questions over the internet. However, many patients were not aware of this, and only 5% of the Dutch patients sent a message to their HCP through a Web-based service [13,14].

The current Dutch health care information landscape entails a variety of institution-based patient portals that are not connected with each other, reflecting the fragmented health care system. In the health care system, the general practitioner (GP) has a central position and functions as a gate keeper for most diagnostic services and specialist care. When referred by their GP, patients can visit several diagnostic centers and see HCPs in several hospitals. Test results, diagnoses, care plans, and progression of treatment are generally reported back to the GP. Direct exchange of information between medical specialists from different hospitals is less common.

### Objectives

Now that more and more health care organizations are implementing patient portals, patients are likely to come across a number of different patient portals that allow them access to personal health information in their records and offer opportunities to exchange secure messages with their HCPs. Theoretically, this positions them in the center of communication concerning their health, together with their GP. Therefore, the implementation of patient portals creates opportunities for patients to play a more active role in decision making, management of their health conditions, and coordination of their health care [5,15-17]. However, it is not clear how patient portals are used by patients and HCPs in the Dutch setting. To expand our knowledge of the implementation of institution-based patient portals in a fragmented health care system, we explored the adoption, utilization, usability, and usefulness of one of the first patient portals in an academic hospital in the Netherlands, 1 year after its introduction.

## Methods

### The Patient Portal

The patient portal under study is a version of MyChart, the patient portal of Epic Systems (Epic Systems Corporation). The functionalities of the portal that could be used during the study period were the list of diagnoses, list of prescribed medications, letters addressed to the GP, laboratory results, and functionalities to schedule appointments and send messages to HCPs. When sending a message, patients are informed that they should receive a response within 2 weeks. After triage by a nurse, HCPs receive the messages in their in-basket, which is not integrated with their email inbox. HCPs are instructed to answer the messages within 1 week. During the study period, the portal could also be used for filling out disease-specific, preconsultation questionnaires and patient satisfaction surveys.

The portal was introduced in 2015. Since then, flyers and banners in the waiting rooms of the hospital and the hospital website have been used to promote the portal as an additional hospital service aimed at patient convenience. In addition, all new patients have been invited to sign up for the portal upon registration with the hospital. After face-to-face identification at the hospital registration desk, patients receive an activation code for opening an account with the portal. The hospital website offers basic instructions on how to use the portal. In addition, patients can get in-person support from the help desk during office hours. All hospital staff received a 4-hour instruction for using Epic, which did not include a formal training in using the patient portal.

### Study Design

The conceptual framework of the study was based on the *Unified Theory of Acceptance and Use of Technology* (*UTAUT*) [18]. The UTAUT relates adoption of a new technology to performance expectancy of the new technology (perceived usefulness), effort expectancy (perceived usability), social influence, and other facilitating factors [18]. We included the constructs of the UTAUT in a multilevel mixed methods study. At patient level, quantitative data were collected regarding utilization and user characteristics. In addition, qualitative data were collected regarding usability and usefulness, facilitating factors, and social influence. At HCP level, only qualitative data were collected on how the portal was used by HCPs (facilitating factors and social influence for adoption by patients) and user experiences (usability and usefulness). Patient and hospital staff qualitative data were first analyzed separately and subsequently compared in a framework analysis. Quantitative and qualitative findings were interpreted together, using the qualitative findings to explain the quantitative results. Textbox 1 presents the study design.

**The multilevel mixed methods study design.**

Patient level quantitative study arm

- **Outcomes:** user characteristics, portal adoption, use of functionalities
- **Data collection:** hospital information system data, log data
- **Analyses:** descriptive, logistic regression
- **Interpretation:** qualitative findings are used to explain quantitative findings

Patient level qualitative study arm

- **Topics:** perceived usability, perceived usefulness, social influence, facilitators for portal use
- **Data collection:** think aloud observation, focus groups
- **Analyses:** thematic content analysis, framework analysis of patient and health care provider (HCP) data together
- **Interpretation:** qualitative HCP findings are used to explain qualitative patient findings

HCP level quantitative study arm

- **Topics:** perceived usefulness, social influence, facilitators for portal use
- **Data collection:** focus groups
- **Analyses:** thematic content analysis, framework analysis of patient and HCP data together
- **Interpretation:** qualitative patient findings are used to explain qualitative HCP findings

### Setting and Participants

The study was conducted at the Radboud University Medical Center, one of the 8 academic hospitals in the Netherlands. This was one of the first hospitals that launched a patient portal in the Netherlands. In 2013, a less advanced portal with fewer functionalities had been introduced, which was replaced by the MyChart portal when Epic was implemented in the hospital in 2015.

The quantitative study arm included patients who were registered on the portal between January 1, 2016, and January 1, 2017, and patients older than 12 years who had visited the hospital at least once in the year 2016 and were alive on December 31, 2016. Think-aloud participants were recruited through referrals from the hospital registration desk. The focus group participants (patients and HCPs) were recruited from 2 outpatient departments: the dermatology and ophthalmology departments. These departments were selected because of the differing portal adoption rates among patients and the divergent attitudes toward the portal among the staff of these departments. The adoption rate of the portal was 25% among patients of the dermatology department and 15% among patients of the ophthalmology department. In an informal qualitative assessment by the information technology department of the hospital, the staff of the dermatology outpatient department was most enthusiastic about the portal, whereas the staff of the ophthalmology department was least enthusiastic of all outpatient departments of the hospital.

For the 2 focus groups with patients, a randomly selected sample of 184 adult patients who had used the portal at least once between September 1, 2016, and February 28, 2017, was invited by their HCPs. For the 2 focus groups with HCPs, hospital staff who had been working at the departments during the last 3 months of 2016 and who had some experience using the portal were invited to participate. To gain a broad perspective on HCPs’ experiences with and attitudes toward the portal, we applied a purposeful sampling strategy including all relevant HCPs, that is, doctors, nurses, assistants, administrative staff, and managers. Participants of the think-aloud observations and focus groups were asked to sign for their consent at the beginning of the sessions. Participating patients received a gift card of €20, whereas HCPs were not compensated for their participation. Ethical approval was requested and granted by the Research Ethics Committee of the Radboud University Nijmegen Medical Centre under number 2016-3091.

### Data Collection

For the quantitative study at patient level, log data were collected from all portal users in the year 2016, including the number of times they had logged on to the portal and the functionalities they had used. In addition, we collected data from the hospital information system on all patients older than 12 years (portal users and nonusers) who visited the hospital at least once during 2016, including sex, age, number of open medical specialist trajectories, and number of open diagnoses. For socioeconomic status (SES), we used the status scores for postal code zones provided by the Netherlands Institute for Social Research for the year 2014. These scores are based on income, employment, and education levels of the inhabitants in the postal code zones [19]. Higher scores refer to higher SES.

We used think-aloud observation and inquiry to assess usability and user-friendliness of the portal while avoiding recall bias [20,21]. Patients without previous experience with the portal were asked to perform a number of predefined tasks, including logging in to the portal, booking a fictitious appointment, checking test results and new messages, and navigating to questionnaires. They were instructed to *think aloud* or verbalize their thoughts while performing these tasks. After completing the tasks, they were asked to reflect on their experiences. To gain further insight into the patients’ reasons to use the portal, experiences with the portal, and their opinions concerning its usefulness, potential benefits, and drawbacks, we organized 2 focus group discussions with patients who were familiar with the portal. In addition, we conducted 2 focus groups with hospital staff to explore their experiences and opinions as well. All focus groups were guided by 2 moderators (SR, MH, YdM, or RBK) who used predefined topic lists, based on the constructs of the UTAUT. The lists included reasons to use the portal, experiences with the portal in general and with the different functionalities, and opinions about the usability and usefulness of the portal and how the portal might be improved. In addition, participants were invited to deliberate about facilitating factors for adoption of the portal and the specific functionalities and the role the portal had or could have in the care processes. The potential impact of the portal was also included as a topic. All think-aloud sessions and all focus group discussions were audiotaped.

### Analysis

#### Analysis of Quantitative Data

Descriptive statistics were used to analyze portal users’ gender, age, number of diagnosis-treatment combinations, number of treatment programs, and SES. We compared the characteristics of portal users and nonusers using a 2-sample *t* test (mean age), a chi-square test (sex), and the nonparametric Kolmogorov-Smirnov test (number of open diagnoses, SES score, and number of medical specialist treatment trajectories). Logistic regression was used to analyze which characteristics were associated with having logged in to the portal at least once, stratified for sex. The covariates included in the logistic regression analyses were the number of medical specialist trajectories (dichotomous: <1, ≥1), number of open diagnoses (dichotomous: <1, ≥1), SES score (continuous), and age (categorical: <45 years, 45-75 years, and >75 years). Age was included as a categorical variable as we expected a nonlinear relation between age and portal use, based on the distribution of age among users, which demonstrated 2 peaks, one around the age of 30 years and another around the age of 60 years, and a steep down slope at the age of 75 years. We used descriptive statistics for the analysis of the frequency of use of the portal in general and the specific functionalities. All analyses were performed using SAS Enterprise Guide.

#### Analysis of Qualitative Data

All think-aloud observations and focus group discussions were transcribed verbatim. We performed a thematic content analysis primarily using an inductive approach and secondarily using a deductive approach with a focus on the constructs of the UTAUT (usability, usefulness, social influence, and other facilitating factors) and the HCP-patient relation and roles. Overall, 3 researchers (SR, MH, and YdM) coded the transcripts independently using ATLAS.ti. After having coded the first think-aloud session and the first focus group discussion, the team discussed codes until consensus was reached and the code book was composed. This was used for coding the other sessions. After all sessions were coded, subthemes were identified for the focus group discussions among HCPs and patients and the think-aloud observations. After discussion with the complete research team, the subthemes were entered in a matrix to enable a thematic content analysis using framework methodology [22]. The matrix displayed the subthemes for HCP focus groups, patient focus groups, and think-aloud observations, together with the codes, and for each code, the most representative quote (see Multimedia Appendix 1). The matrix was used to identify overarching themes by 3 researchers (TV, SR, and RBK), combining the findings from the 3 qualitative study arms.

## Results

### Quantitative Results

In 2016, 24,514 out of 181,679 patients, 13.49% of all patients who visited the hospital, used the portal at least once. Users and nonusers of the portal did not differ with respect to gender, 54.80% of users were female versus 54.49% of nonusers (*P*=.29). The mean age of portal users (50.8 years) was slightly higher than the mean age of nonusers (50.3 years). Users had a higher median SES score than nonusers (0.26 vs 0.18; *P*<.001). More open diagnoses were registered for portal users than nonusers (median: 2.0 vs 1.0; *P*<.001; Table 1).

In 2016, a total of 28,419 patients logged in to the portal at least once. The mean number of times that portal users logged in to the portal during that year was 13 (interquartile range 3-16, mode 1). Logistic regression analyses demonstrated that in both females and males, portal usage was associated with belonging to the age group 45 to 75 years, having a higher SES, having at least one medical specialist trajectory, and having at least one open diagnosis (Table 2). For females, the effect of having 1 or more open diagnoses on portal use was stronger in the youngest and oldest age groups than in the age group 45 to 75 years. For males, there was an interaction between the effects of having a trajectory with a medical specialist and having 1 or more diagnoses.

Most patients who logged in to the portal checked their laboratory results (89.7%), the incoming messages (88.9%), the letters to their GP (87.5%), and their appointments in the calendar (87.4%). The medication list was viewed by 42.6% of the portal users. Fewer portal users sent a message through the portal (20.5%) or used the portal to book an appointment (1.8%).

**Table 1:** Characteristics of portal users versus nonusers.

Characteristics	Patients who visited the hospital in 2016 (N=181,679)	Users of the portal (N=24,514)	Nonusers of the portal (N=157,165)	*P* value
Females, %	55.90	54.80	54.49	.29^a^
Age (years), mean (SD)	50.4 (19.6)	50.8 (16.8)	50.3 (20.0)	<.001^b^
Number of medical specialist trajectories, median (range)	1 (1-16)	1 (1-15)	1 (1-16)	<.001^c^
Number of open diagnoses, median (range)	1 (0-28)	2 (0-23)	1 (0-28)	<.001^c^
Socioeconomic status score, median (range)	0.19 (−5.38 to 3.02)	0.26 (−5.38 to 3.02)	0.18 (−5.38 to 3.02)	<.001^c^

^a^χ^2^ test.

^b^2-sample *t* test.

^c^Kolmogorov-Smirnov test.

**Table 2:** Logistic regression analysis for females and males with dependent variable use of the portal.

Covariates		Model A			Model B		
		Regression coefficient (SE)	OR^a^ (95% CI)	*P* value	Regression coefficient (SE)	OR (95% CI)	*P* value
**Females**							
	Age group 45-75 years^b^	0.5320 (0.0193)	1.13 (1.09-1.17)	<.001	0.5489 (0.0199)	1.73 (1.66-1.80)	<.001
	Age group >75 years^b^	−0.9427 (0.0338)	0.26 (0.23-0.29)	<.001	−0.9384 (0.0350)	0.39 (0.20-0.77)	<.001
	≥1 diagnosis	0.4851 (0.0163)	2.64 (2.48-2.81)	<.001	0.4847(0.0353)	1.62 (1.51-1.74)	<.001
	≥1 medical specialist trajectory	0.1062 (0.0165)	1.24 (1.16-1.32)	<.001	0.1071 (0.0347)^c^	1.11 (1.04-1.19)	.001
	SES^c^	0.1055 (0.0099)	1.11 (1.09-1.13)	<.001	0.1065 (0.0099)	1.11 (1.09-1.13)	<.001
	≥1 medical specialist trajectory, age group 45-75 years	—^d^	—	—	0.1487 (0.0376)	1.16 (1.08-1.25)	<.001
	≥1 medical specialist trajectory, age group >75 years	—	—	—	−0.0455 (0.0667)	0.96 (0.84-1.09)	.47
	≥1 diagnosis, age group 45-75 years	—	—	—	−0.1966 (0.0381)	0.82 (0.76-0.88)	<.001
	≥1 diagnosis, age group >75 years	—	—	—	0.0242 (0.0679)	1.02 (0.89-1.17)	.65
**Males**							
	Age group 45-75 years^b^	0.5068 (0.0171)	1.72 (1.64-1.81)	<.001	0.5072 (0.0276)	1.72 (1.64-1.81)	<.001
	Age group >75 years^b^	−0.4723 (0.0273)	0.65 (0.59-0.71)	<.001	−0.4732 (0.0171)	0.65 (0.59-0.70)	<.001
	≥1 diagnosis	0.3344 (0.0204)	1.95 (1.80-2.11)	<.001	0.3748 (0.0025)	1.45 (1.44-1.46)	<.001
	≥1 medical specialist trajectory	0.1958 (0.0205)	1.48 (1.37-1.60)	<.001	0.1558 (0.0250)	1.12 (1.07-1.18)	<.001
	SES	0.1053 (0.0112)	1.11 (1.09-1.14)	<.001	0.1053 (0.0112)	1.11 (1.09-1.14)	<.001
	≥1 medical specialist trajectory, ≥1 diagnosis	—	—	—	0.0788 (0.0250)^c^	1.08 (1.03-1.13)	<.001

^a^OR: odds ratio.

^b^Reference age group <45 years.

^c^SES: socioeconomic status.

^d^Not included in model A.

### Qualitative Results

A total of 8 patients were recruited to participate in the think-aloud observations, 5 males and 3 females. The ages ranged from 21 to 71 years, with a median of 59 years. They differed in education level and in self-reported digital skills (Table 3). One of the participants got confused when trying to log in to the portal and failed to log in even with encouragement and assistance from the researcher. The think-aloud observations lasted approximately 20 min.

**Table 3:** Characteristics of all participants of the qualitative study arms.

Characteristics		Think-aloud observation participants (N=8)	Focus groups patients (N=12)	Focus groups hospital staff (N=17)
**Sex, n (%)**				
	Male	5 (63)	7 (58)	5 (29)
	Female	3 (38)	5 (42)	12 (71)
Age (years), median (range)		59 (21-71)	63 (34-79)	46 (23-64)
**Education level, n (%)**				
	Low	None	None	—^a^
	Medium	4 (50)	5 (42)	—
	High	3 (38)	6 (50)	—
	Unknown	1 (13)	1 (8)	—
Self-reported digital skills (1: very bad, 10: excellent), median (range)		7 (5-10)	7 (5-10)	8 (7-9)
**Position (hospital staff), n (%)**				
	Medical specialist	—	—	3 (17)
	Medical specialist in training	—	—	4 (24)
	Nurse	—	—	4 (24)
	Doctor’s assistant	—	—	1 (6)
	Administrative employee	—	—	3 (18)
	Manager	—	—	2 (12)

^a^Not applicable.

Overall, 7 male and 5 female patients aged between 34 and 79 years participated in the focus groups. Their education levels were medium or high and their self-reported digital skills ranged from mediocre to excellent (Table 3). Furthermore, 3 medical specialists, 4 medical specialists in training, 4 nurses, 3 administrative assistants, 1 doctor’s assistant, and 2 managers of the outpatient departments participated in the focus group discussions that were conducted with hospital staff. The characteristics of all participants of the qualitative study arms are summarized in Table 3.

The subthemes that we distinguished from the think-aloud observations and all 4 focus groups were adoption of the portal by patients and HCPs; stimulating use of the portal; learning to use the portal; available support for using the portal; procedure to log on; understandability of the information in the portal for patients; functionalities, benefits, patient engagement, and control; patient-HCP relationship; work process; and care process (see Multimedia Appendix 1). Analysis of the matrix resulted in the identification of 5 overarching themes: (1) usability and user-friendliness of the portal, (2) HCP-patient communication through the portal, (3) usefulness of the information that can be accessed through the portal, (4) integration of the portal in care and work processes, and (5) patient and doctor roles and relationships.

#### Overarching Theme 1: Usability and User-Friendliness of the Portal

The think-aloud observations and the focus group discussions revealed some difficulties concerning the log in procedure. However, once logged in to the portal, the layout and the drop-down menus were appreciated, and there were no difficulties to navigate to the different pages:

I think if you have looked at mijnRadboud a couple of times, you know how it works.

Patient 7

Although the think-aloud participants succeeded in booking a fictitious appointment, some focus group patients mentioned that they were not able to do so.

In the focus groups of the HCPs, concerns were raised about the understandability of the information that patients could access through the portal:

If I write an ophthalmologic report, even other medical specialists ask me: what do you mean. Therefore, my reporting cannot directly be translated into information that patients can understand.

HCP 3

However, in the patient focus groups, this was discussed as a surmountable problem. Patients pointed out how they found explanations for what they did not understand on the internet or with their family or friends:

Well, sometimes you need to search on the internet (...) sometimes you see terminology and you think: What are they talking about? But usually it is easy to find out.

Patient 2

Patients appreciated that test results were provided with reference values as it helped them to interpret the results. Regarding the full histological reports, it was mentioned that these were difficult to understand and that the conclusion would be sufficient:

Just report the final result. What needs to be done.

Patient 5

#### Overarching Theme 2: Health Care Provider–Patient Communication Through the Portal

Both HCPs and patients expressed uncertainties about the new opportunities for communication that the portal provides. There was disagreement among the HCPs who attended the focus group discussions on how the messaging functionality should be used. Nurses and administrative employees reported that they advised patients to ask their questions through the portal. In contrast, some doctors commented that they preferred not to use the messaging functionality of the portal to answer questions, especially when it concerned complex problems:

Sometimes the messages are so complex that a telephone call should be scheduled. However, we are not so well organized yet, and not all doctors agree on this.

HCP 16

We try to limit the number messages, because it takes so much time, or it may cause misunderstandings or conflicts.

HCP 1

Patients appreciated that they could ask questions in between consultations through the portal:

That you can ask your questions. That you do not need to call, that you do not need to wait. That you get an answer within a certain period of time. I find that great.

Patient 10

Uncertainties about sending messages were also mentioned in the focus groups with patients. It was not always clear who would read the messages:

(It says) You can ask questions to your team. Then I think: Who are all these people? To whom do I send this question?

Patient 11

In addition, there were uncertainties about the kind of questions and the number of questions that might be included in a message:

I felt hesitant because I had very many questions and because it was new to me.

Patient 9

Some patients also mentioned feeling reluctant sending messages because of concerns about the workload of the doctors:

I do not want to bother my doctor with this, he is much too busy.

Patient 11

Among the HCPs, there were reservations concerning patients’ access to the letters to the GP. On one hand, it was recognized as an additional way to communicate with their patients:

It helps me sometimes as well, if I know that a patient reads it (the letter to the GP). It gives me another opportunity to show how thorough I am. That I can say: We have done this and that, we have excluded that, it is not cancer.

HCP 6

On the other hand, there were concerns that this might limit the original function of the letters to report relevant medical information to the GP:

I also notice it when writing the letters to the GP. Then I think, the patient reads this as well. Then I formulate more cautiously and I just hope that the GP will still understand what I mean.

HCP 3

The experiences of HCPs with the questionnaire functionality differed. On one hand, they were considered a useful time-saving tool:

The alternative is that someone walks in with a (paper) questionnaire and that I have to go over it in 5 or 10 minutes. While now, it (the filled-out questionnaire) is just there, with one press of the button. Then I check it and it is done.

HCP 1

On the other hand, there were concerns that the information from the questionnaires was not accurate:

If the patient fills something out, and does not understand the question correctly, then you don’t know. Then he fills something out that is not correct.

HCP 2

In the focus groups with patients, the questionnaire functionality was not discussed.

#### Overarching Theme 3: Usefulness of the Information That Can Be Accessed Through the Portal

Some patients were satisfied with the amount and type of information they could access through the portal, whereas others would like to be able to view more information. Patients appreciated being able to view their test results, especially for monitoring their conditions:

Another advantage is that you can compare the lab results with those from a few months ago. You can see whether they have gone up or down, are they better or not.

Patient 5

Although patients appreciated to have timely access to their results, some reservations about receiving potentially sensitive information through the portal were put forward:

Something I did not like was when I had had a biopsy. Then you have the results before you have seen the doctor. I googled it and found out what it meant. Not exactly of course, because I am not a doctor. I think that the doctor should discuss the results before you can see them through the portal.

Patient 5

The HCPs agreed with this, emphasizing that informing patients about test results is their responsibility and that they did not want their patients to worry unnecessarily about their results when viewing them without explanations:

You are medically responsible and you prefer the patient not to see the results before you, the professional, sees them.

HCP 6

Notwithstanding the uncertainties about how to use the secure messaging functionality, there was appreciation among patients for this functionality as an opportunity to formulate their questions more accurately and ask them in between visits:

I can ask my questions when they come up. (...) I can take time to formulate my questions, re-read them to check if I put it right and then send them. During visits, you are not sure whether you ask the right question. Afterwards you think: I should have asked something else.

Patient 6

Patients also appreciated being able to check the calendar as a reminder for upcoming appointments.

The medication list was considered less useful as this was not always up to date. In contrast, access to the letters of GPs was considered useful by patients as it provided them with a summary of what was discussed:

For me the great advantage is that you can read what was decided. You do not need to remember it, it is just there.

Patient 3

#### Overarching Theme 4: Integration of the Portal in Care and Work Processes

Patients noticed that the use of the portal for care processes varies among HCPs:

There are some (HCPs) that really use it. For example, the nephrologist. He says, check your blood pressure and send the results. That stimulates. There are other departments where the portal is hardly mentioned at all.

Patient 7

HCPs mentioned that some colleagues do not want to use the portal:

There is a group (of medical specialists) who is fundamentally against these sort systems. Also, against electronic health records and all sorts of innovations in this hospital.

HCP 5

Patients reported using the portal for preparation of their consultations as a reminder of what was discussed during visits and of upcoming appointments. In addition, they used the portal to contact their HCP in between visits, thus using it as a continuation of their contact with their HCPs and regular care:

If you have read it beforehand, you can ask the right questions. (...) That is much more efficient, also for the doctor.

Patient 6

In addition, HCPs felt that the consultations might improve when patients use the portal:

They are better informed about their medical history, which makes it easier to ask questions during consultations (...) I think this may help during consultations.

HCP 17

According to the HCPs, the portal was not firmly integrated in the work process of HCPs yet:

What we don’t do very well yet, I suppose because the impact is not substantial yet, is that we do not adjust our work processes to the portal.

HCP 1

Answering the messages was not formally incorporated in the daily work process of doctors:

There is an extra work load because of the in-basket messages, which seem to be sent with little reluctance. (...) You need to plan extra time to process these messages.

HCP 3

Some HCPs mentioned that they answered messages in their spare time.

#### Overarching Theme 5: Doctor and Patient Roles and Relationships

Some patients reported feeling more engaged in their health care and having more control by using the portal:

I feel that I have a little more control. Before I depended on the GP or the medical specialist. Now I can monitor the results myself and I like to be able to do that.

Patient 4

Others did not think that their relationship with the HCP had changed after they had started to use the portal:

No, it has not changed the relationship because there is not enough information in it. Only appointments and letters to the GP. So, you can’t refer to it, like “Doctor I read this and that...”

Patient 10

HCPs noted changes in the role patients played in their care, using the portal for checking their test results before visits:

I had a patient who said, the ALAT (alanine aminotransferase) has increased, is that a problem doctor? That was a nice question (...) I liked the conversation. Before, you did not have these types of discussions (...) it changes the dynamic.

HCP 5

They also mentioned changes in their role related to the use of the portal:

It feels a little uncomfortable. It feels as if you lose your autonomy as a doctor, because you have your partner, the patient in this case, sitting next to you. Personally, I think this is a good development; I suppose we have to get used to it.

HCP 5

HCPs also felt that they lost some control over the health care and communication processes by allowing patients to reschedule appointments through the portal and by providing patients the possibility to send 24×7 messages without any restrictions.

## Discussion

### Principal Findings

One year after the implementation of the patient portal, 1 out of 7 patients who visited the hospital logged on to the portal. Predictors for adoption were having at least one diagnosis or a medical specialist trajectory open in the hospital, having a higher SES, and belonging to the age group 45 to 75 years. Patients mainly used the portal to view their laboratory results, incoming messages, and letters to their GP. Qualitative analyses revealed how these functionalities could be useful for patients to increase their engagement in their care, that is, to monitor their condition, to remember what was decided during visits, and to prepare for consultations. In addition, patients described how the portal might make their interactions with the hospital more convenient, for example, using the calendar and asking questions in between visits. Regarding the use of the portal for patient-HCP communication, uncertainties were raised by patients and HCPs about what type of questions and how many questions might be asked or answered through the portal. These uncertainties might explain the rather low utilization of secure messaging functionality. Patients and HCPs noted that the use of the portal was not integrated in care and work processes in all the departments of the hospital. Regarding the potential impact of the portal on patient and HCPs roles, potential loss of autonomy and control over care and communication were brought up by HCPs. Although some patients mentioned the feeling of having more control, others did not find that the portal had changed the relationship with their HCP substantially.

### Relation With Findings From Other Studies

#### Adoption of the Portal

To our knowledge, this study is the first to report an adoption rate of a patient portal in a real-world setting in the Netherlands. When compared with international studies, the adoption rate of 13.5% corresponds to the mean adoption rate of 23% (95% CI 13%-33%) for patient portals in real-world studies reported in a systematic review and meta-analysis on portal adoption [23].

Similar to the results of some reviews on patient portal adoption [24,25], we found an association between portal adoption and belonging to the middle-aged group and having a higher SES. However, in our study, the older age group, being over 75 years, was older than the older age group in most of the studies that were included in these reviews. Lower adoption rates among older patients and patients with lower SES have been explained by limited access to the internet, limited skills to use the internet, limited health literacy, and concerns about security and privacy among older and less educated patients [24-27]. Our study did not include these variables, but limited access to the internet is likely to have contributed to lower adoption among patients older than 75 years in our study. In the Netherlands, in 2016, the year this study was conducted, access to the internet was much lower among people older than 75 years as opposed to the younger age groups, 60% versus 90% to 99% [28].

The other predictors of portal adoption that we found, having at least one open medical diagnosis or trajectory with a medical specialist rather than being a 1-time visitor to the hospital, may point at a higher perceived relevance of the portal for patients with a (chronic) disease and frequent users of health services, as suggested by some reviews [2,23,25]. However, frequent visitors of the hospital may also be more aware of the portal as they are more likely to come across the banners and the flyers about the portal.

#### Usability of the Portal

Limited user-friendliness or usability problems have been described as barriers for portal adoption [29]. We found that, apart from the challenging procedure to log in, usability problems do not seem to have hampered adoption and continuing use. Patients found the portal easy to navigate and also explained that they understood most of the information, including medical terminology, if necessary, with help from their family, friends, or the internet. This corresponds with the results of the OpenNotes study that demonstrated that few patients reported not being able to understand the information or reported being confused after reading their visit notes [30]. In addition, a study that investigated the terminologies that patients used in Web-based patient-patient communication suggests that patients are more familiar with the medical terminologies concerning their own health problems than HCPs might be aware of [31]. However, both the OpenNotes study and this study on patient-patient communication did not investigate the patients’ actual understanding of the terminologies and information. In our study, HCPs had concerns about patients not being able to understand the letters to the GP or their test results. These concerns have also been reported in other studies [32], which is not surprising as before the introduction of patient portals and Web-based access to records, the information in the medical records was aimed at professional use. Some HCPs in our study mentioned that they adjusted their reporting to avoid patients getting confused or anxious. Adjusted reporting may not be necessary as patients find explanations from other sources and they may also like to read the medical terminology to learn from this and bring the communication to a level playing field [33,34]. Communicating sensitive information through the portal seemed to be a different issue, not so much related to understanding the terminology, but to the interpretation of the results and putting them in the perspective of the next steps that need to be undertaken regarding further testing or treatment. Among both HCPs and patients, a preference for face-to-face or telephone contact for sensitive results was brought up. In line with this, at the time of our study, there was a delay in presenting test results to provide HCPs the possibility to communicate results to their patients first personally.

#### Usefulness of the Portal

The relevance and usefulness of a patient portal for patients to manage their conditions and health care have been suggested to determine portal adoption and use [2,23]. Our quantitative results that demonstrated an association between portal use and having at least one open diagnosis or at least one medical specialist trajectory, also point at this. Our qualitative results demonstrated that both HCPs and patients who use the portal feel that the portal can help to engage patients in their care, add convenience, and provide a new way of communication between patients and HCP. These issues correspond with the mechanisms through which patient portals have been found to produce effects in a realist review, that is, insight into information and activation of information, patient convenience, and continuity of interpersonal care [4].

Concerning the new channel for communication between patients and HCPs that the portal offers, patients explained how the secure messaging function enabled them to interact with their HCPs in between visits and to reflect on their questions. It has been suggested that patients feel more confident asking questions through secure messaging than during face-to-face contacts and that secure messaging enables patients to set the agenda [35]. Therefore, secure messaging can contribute to patient empowerment and patient-centered communication and eventually have an effect on the power relation between HCP and patients [36]. Our study differs from other studies on portal adoption in the relatively low utilization of the secure messaging functionality by patients [5]. This may be explained by the uncertainties that patients and HCPs have about how to use this functionality and concerns about the workload of HCPs. Similar barriers for using secure messaging have been described by Sieck et al [37]. They emphasize that secure messaging is a new way of communication and therefore requires new rules. They propose to define new *rules of engagement* for patient-HCP communication using secure messaging. In addition, training of patients and HCPs on how to use secure messaging in the care processes have been proposed to overcome these barriers [38-40].

#### Social Influence: Health Care Provider Endorsement and Integration in Care and Work Processes

HCP endorsement of portal use and integration of the portal in care and administrative processes have been found to have a positive effect on portal adoption by patients [23,25]. In terms of the constructs of the UTAUT, HCP endorsement and integration in care processes can be considered as social influence. In the setting of our study, the portal was introduced as an additional service in flyers and on the hospital website. We found that the portal was not embraced equally by all HCPs in the hospital and some HCPs encouraged patients to use the portal, whereas others did not. Furthermore, we found that the portal was not embedded in the care processes in the ophthalmology and dermatology departments. Integration of the portal in care processes will engage patients more with the portal and will help them to use it as a tool for their health care rather than just as an additional service. Integration of the portal in care processes and in the workflow of HCPs is also likely to engage HCPs with the portal, as has been described for the implementation of new technologies [29,41,42]. In addition, it has been argued that in the digital era, HCPs have to become *e-physicians* and need to be empowered to be able to benefit from digital technologies in their work [43].

#### Potential Impact of the Portal on the Patient–Health Care Provider Relationship

Our study was not designed to evaluate the impact of the portal on health outcomes or care processes. The previously mentioned realist review linked the mechanisms through which patient portals have been found to work (insight into information and activation of information, convenience, and continuity of interpersonal care) to the effects on health outcomes, patient empowerment, adherence, patient satisfaction, communication, and the patient-HCP relationship [4]. Of these potential effects, the patient-HCP relationship was discussed in the focus groups. Some HCPs felt changes in their control over care processes and also patients mentioned the feeling of having more control. Neither mentioned substantial changes in their roles or in relationship, in the sense of this getting better or worse or more equal. This may be because it was still early to observe the effects on the HCP-patient relationship. However, it may also have to do with the low use of the secure messaging function and the limited integration in care processes or the limited information that can be accessed through the portal. Furthermore, some patients and HCPs pointed out that the portal plays only a small role in their interactions.

### Strengths and Limitations

A strength of this study is the multilevel mixed methods design. Mixing qualitative think-aloud observations and focus group discussions among patients and HCPs with quantitative log data and user characteristics helped to deepen our insight into portal utilization. However, the obtained insight may not be complete as we did not include patients who did not use the portal in the focus group discussions. Therefore, we do not have information on other barriers for adoption of the portal, other than the challenging procedure to log in to the portal. In addition, we did not collect individual data on health literacy and education level, variables that are likely to be of more importance for predicting portal adoption than SES score, which is a score at neighborhood level. Another limitation is the selection of the departments from which we recruited patients and HCPs for the focus group discussions. As we expected, the HCPs in the dermatology and ophthalmology departments differed in their utilization of the portal, and thus provided us a broad range of experiences and opinions. However, this choice may have limited the range of patient experiences that we were able to identify. To obtain more variation in patient experiences and opinions, especially regarding engagement in care and self-management, it would have been interesting to include a department where more patients with chronic diseases were treated, for example, the nephrology or oncology department.

### Implications for Clinical Practice

This study suggests that the adoption and use of a patient portal might improve if patients and HCPs are informed about how the functionalities of the portal should be used. In addition, use and usefulness of patient portals may improve if HCPs incorporate the portal in their care practices. They can, for example, explain patients how to adjust medication after certain test results or ask patients to prepare for consultations by looking at their test results before a face-to-face consultation. They also might encourage patients to share information that they can access through the portal with their HCPs from other health care organizations and stimulate them to play an active role in the coordination of their care in the fragmented health care system. Hence, HCPs can contribute to a portal being more than just a new service and make it a valuable instrument to improve health care. Regarding the secure messaging functionality, the hospital and individual HCPs should be clear about what sort of questions can be asked through the portal and who will read the messages. In addition, HCPs should be aware that elderly patients and patients with lower SES scores are less likely to use the portal. Furthermore, the HCPs should be allowed to reserve time in their schedules for answering the messages.

### Further Research

We have used the constructs of the UTAUT, perceived effort, perceived usefulness, and social influence to investigate the adoption and use of the portal. We included HCP endorsement and integration in care and work processes in our study as social influence, which provided us some insight into the complexity of portal adoption. However, the UTAUT may not be the best model to investigate the complex interplay among the HCPs, between HCPs and patients, HCPs and the hospital, and patients and the hospital related to portal adoption and nonadoption. Recently, Greenhalgh et al proposed a framework for theorizing and evaluating nonadoption, abandonment, scale-up, spread, and sustainability of health and care technologies (NASSS) [44]. The NASSS framework includes the following 7 domains: the condition, the technology, the value proposition, the adopter system (HCPs, patients, and caregivers), the health organization, the wider institutional and societal context, and the adaptations over time. The NASSS framework overlaps partly with the UTAUT but uses a broader and less linear perspective on adoption. Therefore, it may be useful to use this framework instead of the UTAUT in future studies.

We found that portal adoption may have been hampered by limited integration in the care and work processes. Therefore, implementation research addressing how to embed portal use in the care and work processes is called for. Furthermore, as portals provide new ways for HCP-patient communication, research is needed on how to use these new communication tools in relation to other communication tools such as WhatsApp and the conventional telephone calls and face-to-face consultations.

### Conclusions

This study identified some factors that are associated with the adoption of a recently introduced patient portal: age between 45 and 75 years, higher SES, and having an open diagnosis or open trajectory with a medical specialist. It demonstrated that patients and HCPs recognize the potential of a patient portal to engage patients in their care processes, facilitate patient-HCP communication, and increase patient convenience. Limited integration of a patient portal in care is likely to limit its adoption by patients and its usefulness for patient engagement. Vague and inconsistent information about how to use the new communication opportunities of a portal are likely to hinder the utilization of the communication functionality. Instructions on how to use the functionalities of a portal and integration of a portal in care and work processes may improve the utilization of a patient portal and are likely to contribute to a portal becoming a more valuable tool for improving patient engagement, HCP-patient communication, and patient convenience.

## Appendix

Multimedia Appendix 1 Matrix qualitative data.

## Abbreviations

GP: general practitioner
HCP: health care provider
NASSS: nonadoption, abandonment, scale-up, spread, and sustainability of health and care technologies
SES: socioeconomic status
UTAUT: Unified Theory of Acceptance and Use of Technology
