# Supplementary material for: Patient and Health Care Provider Experiences With a Recently Introduced Patient Portal in an Academic Hospital in the Netherlands: Mixed Methods Study
Source: J Med Internet Res. 2019 Aug 20;21(8):e13743. doi: 10.2196/13743 (PMC6788335; doi:10.2196/13743)
Supplement: Multimedia Appendix 1 [file jmir_v21i8e13743_app1.pdf]

	**Focus groups HCPs**<br>**Codes and quotes**<br>(D=doctor, O=other HCP(nurse, manager, administrative worker))	**Focus groups patients**<br>**Codes and quotes**<br>(Pt=patient)	**Think aloud observations patients**
**Adoption by patients**	▪ Limited use by patients<br>*“Once in a while you see someone who has viewed a letter and they are quite satisfied. It does not have a great impact on the daily practice.”(D1)*	▪ Adoption because of lab results<br>*“I started using mijnRadboud because I like to view some of my results”(pt4)*<br>▪ Possibility to ask questions<br>*“Until recently it was only annoying. Only useful for appointment reminders, but you get those also through e-mail. But then I discovered that I could ask questions. That was a pleasant addition.” (Pt12)*	
	▪ Increasing use by patients<br>*“We notice that we get more questions about what patients have read through the portal, especially lab results.” (D5)*	▪ Adoption because of letters<br>*“I like to read the letters that are sent”(pt3)*	
**Stimulating use of the portal**	▪ Patient as driver for adoption of the portal among HCPs<br>“*The patient is the one who should be guiding (the adoption process of the portal among HCPs) If the patient says that he wants it, who is the doctor to say that he won’t.” (D5)*	▪ Information about the portal for patients<br>*“I walked past (a banner) and thought ‘hey that is interesting’ Then I looked it up at google at home. And the next time I had to be here I registered for it. “ (Pt10)*	
	▪ Recommending patients to use the portal<br>“*I recommend it (the portal), but I explain that we are working to improve it. Otherwise they lose interest.” (O1)*	▪ Utilisation stimulated by HCP<br>*“There are some that really use it. For example the nephrologist. He says, check your blood pressure and send the results. That stimulates. There are other department where the portal is hardly mentioned at all.” (Pt7)*	
**Adoption by HCPs**	▪ Colleagues not in favour of innovations<br>*“There is a group (of medical specialists) who is fundamentally against these sort systems. Also against electronic health records and all sorts of innovations in this hospital” (D5)*	Differences in adoption<br>*“There are other departments, where the portal is hardly mentioned at all..” (pt7)*	

	▪ Uncommitted use of the portal<br>*“There is still room for uncommitted use of the portal, in the use of the in-basket” (O9)*	▪ HCPs do not know the portal<br>*“My doctor did not even know (...) she knew nothing about mijnRadboud”(pt11)*	
**Learning to use the portal**	▪ New issues<br>“*We still get new issues, that we have not had before. We still need to learn from these and adjust our work processes. New issues. More and more patients. New types of questions.***”** *(O6)*<br>▪ Instruction might be useful<br>“*What would be very helpful for us, is an e-learning about the portal.” (O10)*	▪ Easy to learn<br>*I think if you have looked a couple of times at mijnRadboud, you know how it works.”(Pt7)*	The general lay-out of the portal with tabs and drop down menus was appreciated.
**Available support for using the portal**	▪ Support for HCPs<br>“*We notice again and again how important technical support is” (D5)*	▪ Limited availability of support<br>*“It says at the website that you can call the information desk (...) but you have to call there at specific times.“(Pt8)*<br>▪ Limited support from HCPs<br>*“My doctor did not even know (...) she knew nothing about mijnRadboud”(pt11)*	
**Procedure to log on**	▪ Colleagues do not know how to use the system<br>“*I think that some colleagues do not even know how mijnRadboud works. They have never logged on.” (O4)*	▪ The procedure to log on works well<br>*“It (*the procedure to log on to the portal*) works very well, but it is another username and password to remember.”(Pt9)*<br>▪ Procedure to log on the portal is complex<br>“Right now, I can’t log on. I need a new password. (...)*Why a password...and codes through the mail, I find that very impractical.” (Pt8)*	One participant failed to log on to the portal, even with help of the interviewer<br>Two patients did not succeed to log on at first because of confusion about the access code and text message verification and because of not recalling the password. When they tried again, they did succeed.
**Understand-ability of the information**	▪ According to HCPs patients do not understand letters<br>“*Patients who have read a letter and then comment on a detail, because they did not understand.”(D6)*	▪ Reference values with test results<br>*“I think it is a plus that they include the reference values with the lab results. You see immediately whether your results are abnormal or not.“ (Pt2)*	One patient indicated that the lab results were difficult to understand

**in the portal for patients**	▪ According to HCPs patients do not understand medical reports<br>*“If I write an ophthalmologic report, then other medical specialists ask me: what do you mean. Therefore, I expect that my reporting cannot directly be translated to useful information for patients” (D3)*	▪ Looking up medical terminology<br>*“Well, sometimes you need to search the internet. Sometimes you come across terminology that you think what are they talking about. But usually you can find it easily.” (Pt2)*	
**Functionality Calendar**		▪ Viewing appointments<br>*“It provides a beautiful overview of your appointments. Appointments that are coming up and appointments that you have forgotten, you can look them up.”(Pt6)*	There were no difficulties in finding the calendar to check upcoming appointments.
	▪ Difficult for patients to book appointments<br>*“I think it is extremely difficult for patients to book an appointment. It is already difficult for us, so how can we expect patients to do this ” (O6)*	▪ Difficulties in booking appointments<br>*“What does not work at all, is booking of appointments” (Pt7)*	Participants were able to book a fictitious appointment.
	▪ Patients booking inappropriate appointments<br>*“It is easy to cancel or reschedule an appointment. That may be convenient for the patient, but sometimes we see a patient, and you think: why do I see him now? Is that really necessary?”(D2)*		
**Functionality Letters to the GP**	▪ Informing patients through letters to the GP<br>*“It helps me sometimes as well, knowing that the patient reads this. I can show again how thorough I am. I explain we have done this and that and excluded that, it is not cancer.” (D6)*	▪ Knowing what the GP knows<br>*“I think it very positive, that I can view the letters that go to the GP, so that I know what the GP knows.”*	Navigating to letters to the GP took most participants some time, but in the end they all found them.
	▪ More cautious in letters to GPs<br>*“Then I think, the patient reads this as well. Then I formulate more cautiously, and then I hope that the GP will still understand what I mean.” (D3)*		
**Functionality Access to lab**		▪ Desire to view lab results<br>*“I started using mijnRadboud because I like to view some of my results”*	All patients were able to navigate to the test results.

**results**	▪ Patients need medical knowledge for interpretation of test results<br>*"You do not want people to worry unnecessarily. That is the problem. Patients have a right to see their results, whatever results. But you need to be medically educated to interpret some results."(D5)*	▪ Interpretation of lab results<br>*"One of the advantages is that you can compare the results with (those of) a few months before. Is there an increase or decrease, is it better or not"(Pt5)*	There were some difficulties in understanding the lab results
	▪ Access to test results with or without delay<br>*"You are medically responsible. And you would rather not have your patients see the results before you, a professional has seen them."(D6)*	▪ Access to test results with or without delay<br>*"What I did not like: I had had a biopsy and then you have the results before you have seen your doctor. I have googled it and then you find out what it is. Not exactly of course, because I am not a doctor. I think that the doctor should discuss the results with you before you can see it through the portal."(Pt5)*	
**Functionality Questionnaire**	▪ Questionnaires can increase efficiency<br>*"The alternative is that someone walks in with a (paper) questionnaire and that I have to go over it in 5 or 10 minutes. While now, it (the information) is just there, with one press of the button. Then I check it and it is done." (D1)*		Four participants did not succeed in finding questionnaires that were open to be filled out.<br>A couple of patients had difficulties checking whether they had sent the questionnaire that they had completed.
	▪ Limited usefulness of questionnaires<br>*"Then you need to check everything and that means duplication of effort" (O3)*		
**Functionality Secure messaging**		▪ Convenient to be able to send messages to HCP.<br>*"That you can ask your questions. That you do not need to call, that you do not need to wait. That you get an answer within a certain period of time. That is just great."(Pt10)*	Most patients found the inbox without difficulties. One patient confused messages with letters to GPs.
		▪ Able to formulate questions correctly<br>*"I can ask my questions whenever they come up. (...) I can take time to formulate my questions , re-read them to check if it is right and then send them. During visits, you are not sure whether you ask the right question."(Pt6)*	

		▪ Timely response to questions<br>*"I must say, you always get an answer very quickly when you ask."(Pt3)*	
	▪ Trying to limit the number of messages<br>*"We try to limit the number of messages, because it takes so much time, it may cause misunderstandings or quarrels."(D1)*	▪ Reluctance to ask questions through the portal<br>*"I do not want to bother my doctor with this, he is much too busy. I also wonder how much may I ask, otherwise it becomes a consultation" (pt11)*<br>*"I felt hesitant because I had very many questions and because it was new to me."(Pt9)*	Some participants mentioned reluctance to ask their doctors questions through the portal because they thought it is a burden for doctors and that doctors may interpret a need for information as a critical question.
	▪ Disagreement between HCPs of when secure messaging or telephone contact is preferable<br>*"Some messages are complex and a telephone consultation should be booked. But not all doctors agree on this."(O10)*		
	▪ Preferable to answer questions over the telephone<br>*"Sometimes people send a message and then they have a long list of questions. Then it is more practical to discuss over the telephone.(D4)*<br>*"But often these questions are complex, you can't just answer them." (D1*		Some participants indicated to prefer contact in person over email contact.
		▪ Not clear to whom to send a message with a question<br>*"You may ask questions to the team. Then I think "Who are these people in my team? To whom do I send this question?"(Pt11)*	
**Functionality Medication list**		▪ The medication list is not up-to-date<br>*"But I find that it is not up to date (...) The medication that I use now for the blood pressure and cholesterol are not included yet."(Pt3)*	

**Information that can be accessed by patients**	▪ Not clear what information is made accessible for patients<br>*“Even for us it is not always clear what patient can and cannot see” And then to explain: it is correct that you cannot see this. It is there, but you cannot see it, because of this and that and that.”(D2)*	▪ The information that can be accessed is limited<br>*“You can view lab results and a summary of what is sent to the GP, but you cannot see what was discussed during consultations”*<br>*“It is not complete, I do want to see results of scans as well.”(Pt4)*	
		▪ The medication list is not up-to-date<br>*“But I find that it is not up to date (...) The medication that I use now for the blood pressure and cholesterol are not included yet.“(Pt3)*	
**Benefits**		▪ Expansion of services that the hospital offers<br>*“For me it adds to the services of the Radboudumc. I think it is perfect. I can do with it that I need to do.”(Pt1)*	All but one of the participants found the portal useful. All but one indicated that they would use the portal again and would recommend it to others.
	▪ Informing patients through the portal<br>*“It helps me sometimes as well, knowing that the patient reads this. I can show again how thorough I am. I explain we have done this and that and excluded that, it is not cancer” (D6)*	▪ To read back what was discussed<br>*“The most important benefit is, that you can read back what was agreed to be done. You don’t need to remember it, it is just there.”(Pt7)*	
	▪ Enabling patients to ask questions<br>*“They are better informed about their medical history, which makes it easier to ask questions during consultations(...) I think this may help during consultations.” (O8)*	▪ Preparation of visits<br>*“If you have read it beforehand, you can ask the right questions. (...)That is much more efficient.“Pt6)*	
	▪ Fewer telephone calls<br>*There are also advantages: fewer telephone calls. I advise patients to ask their questions through mijnRadboud” (O7)*	▪ Possibility to ask questions<br>*“Until recently it was only annoying. Only useful for appointment reminders, but you get those also through e-mail. But then I discovered that I could ask questions. That was a pleasant addition.” (Pt12)*	
	▪ Transparency<br>*“I think, these type of systems make it more transparent for the patient. You show what you have done, the letter that was sent to the GP” (D5)*		

**Patient engagement and control**	▪ It changes the dynamic<br>*"I had a patient who said, the ALAT has increased, is that a problem doctor? That was a nice question (...) I liked the conversation. Before you did not have these type of discussions(...) It changes the dynamic"(D5***)**	▪ More discussions<br>*"I think, because of what you know yourself, you have more discussions with your doctor during visits(...)(Pt6)*	
	▪ Easier to ask questions for patients<br>*"They are better informed about their medical history, which makes it easier to ask questions during consultations(...) I think it may help during consultations." (O3)*	▪ Prepared to ask questions during visits<br>*"You are prepared (...) You do not know exactly what you talk about, this medical terminology, but it is easier to ask questions"(pt5)*	
		▪ More involved<br>*"You are more involved, you have more information." (pt8)*	
		▪ More control<br>*"I feel that I have a little more control. Before I depended on the GP or the medical specialist. Now I can monitor myself and I like to be able to do that."(pt4)*	
		▪ More competent to manage disease<br>*"You become more competent in the management of your condition. Theoretically this helps, but it may also give more uncertainties and more questions" (pt8)*	
**HCP-patient relationship**	▪ Feeling watched by the patient<br>*"It feels as if the patient is watching you closely."(D5)*	▪ No impact on relation<br>*"No, it has not changed the relationship because there is not enough information in it. Only appointments and letters to the GP. So you can't refer to it, like 'Doctor I read this and that...'."(pt10)*	
	▪ Autonomy as a doctor<br>*"It feels a little uncomfortable. It feels as if you lose your autonomy as a doctor, because you have your partner, the patient in this case, sitting next to you. Personally, I think this is a good development; I suppose we have to get used to it" (D5)*		
	▪ Trust<br>*"The conversations change. I think, it is more transparent. Therefore, it is possible to built trust with patients."(D6)*		

**Work process**	▪ Increased work load<br>*“It takes time to answer all these question adequately.” (O7)*	▪ Reluctance to ask questions through the portal<br>*“I do not want to bother my doctor with this, he is much too busy. I also wonder how much may I ask, otherwise it becomes a consultation” (pt11)*	
	▪ More cautious reporting<br>*“You have to be more alert. You must be more careful of what you enter in it (the letter to the GP). You must communicate that with patient beforehand.” (D5)*<br>*“Then I think, the patient reads this as well. Then I formulate more cautiously, and then I hope that the GP will still understand what I mean.” (D3)*		
	▪ Planning time to answer messages<br>*You should plan extra time to process these messages.” (D3)*		
	▪ Adjust work processes to the portal<br>*“What we don’t do very well yet, I suppose because the impact is not substantial yet, is that we do not adjust our work processes to the portal.” (D1)*		
	▪ Getting used to attending patients digitally<br>*“We need to get used to having a new way for attending patients, and that is through the digital counter.(..) That is still difficult, because you do not see the patient. But he is really there.“ (O9)*		
**Care process**		▪ More efficient visits<br>“*If you have read it beforehand, you can ask the right questions. (...)That is much more efficient.“(pt6)*	
		▪ Integration in care process<br>*“For example, the nephrologist, he says: check your blood pressure at home and send it to me. He really stimulates that. There are other departments, where they hardly mention the portal.” (pt7)*	
